# PufQ regulates porphyrin flux at the haem/bacteriochlorophyll branchpoint of tetrapyrrole biosynthesis via interactions with ferrochelatase

**DOI:** 10.1111/mmi.13861

**Published:** 2017-11-17

**Authors:** Jack W. Chidgey, Philip J. Jackson, Mark J. Dickman, C. Neil Hunter

**Affiliations:** ^1^ Department of Molecular Biology and Biotechnology University of Sheffield Sheffield S10 2TN UK; ^2^ ChELSI Institute, Department of Chemical and Biological Engineering University of Sheffield Sheffield S1 3JD UK

## Abstract

Facultative phototrophs such as *Rhodobacter sphaeroides* can switch between heterotrophic and photosynthetic growth. This transition is governed by oxygen tension and involves the large‐scale production of bacteriochlorophyll, which shares a biosynthetic pathway with haem up to protoporphyrin IX. Here, the pathways diverge with the insertion of Fe^2+^ or Mg^2+^ into protoporphyrin by ferrochelatase or magnesium chelatase, respectively. Tight regulation of this branchpoint is essential, but the mechanisms for switching between respiratory and photosynthetic growth are poorly understood. We show that PufQ governs the haem/bacteriochlorophyll switch; *pufQ* is found within the oxygen‐regulated *pufQBALMX* operon encoding the reaction centre–light‐harvesting photosystem complex. A *pufQ* deletion strain synthesises low levels of bacteriochlorophyll and accumulates the biosynthetic precursor coproporphyrinogen III; a suppressor mutant of this strain harbours a mutation in the *hemH* gene encoding ferrochelatase, substantially reducing ferrochelatase activity and increasing cellular bacteriochlorophyll levels. FLAG‐immunoprecipitation experiments retrieve a ferrochelatase‐PufQ‐carotenoid complex, proposed to regulate the haem/bacteriochlorophyll branchpoint by directing porphyrin flux toward bacteriochlorophyll production under oxygen‐limiting conditions. The co‐location of *pufQ* and the photosystem genes in the same operon ensures that switching of tetrapyrrole metabolism toward bacteriochlorophyll is coordinated with the production of reaction centre and light‐harvesting polypeptides.

## Introduction

The purple photosynthetic bacterium *Rhodobacter* (*Rba*.) *sphaeroides* is capable of both aerobic chemoheterotrophy and anaerobic phototrophy (Mackenzie *et al*., 2007). The transition between these growth modes involves invagination of the cytoplasmic membrane, yielding an extensive system of intracytoplasmic membrane vesicles (Tucker *et al*., 2010), often called chromatophores, which house the photosynthetic apparatus. Light energy is absorbed by light‐harvesting (LH)2 complexes, passed to LH1 and in turn to the reaction centre (RC), driving charge separation, generation of a proton‐motive force and eventual generation of ATP (Cartron *et al*., 2014; Sener *et al*., 2016). The RC is a membrane–intrinsic complex composed of L, M and H subunits that sits within a ring of LH1 α and β polypeptides with associated bacteriochlorophyll *a* (BChl *a*) and carotenoid pigments. RC‐LH1‐PufX complexes can form dimers, with the PufX component responsible for dimerisation and for creating a pore in each LH1 ring that allows quinones and quinols to shuttle between the RC Q_B_ site and neighbouring cytochrome *bc*_1_ complexes (Qian *et al*., 2013; Cartron *et al*., 2014).

**Figure 1:**
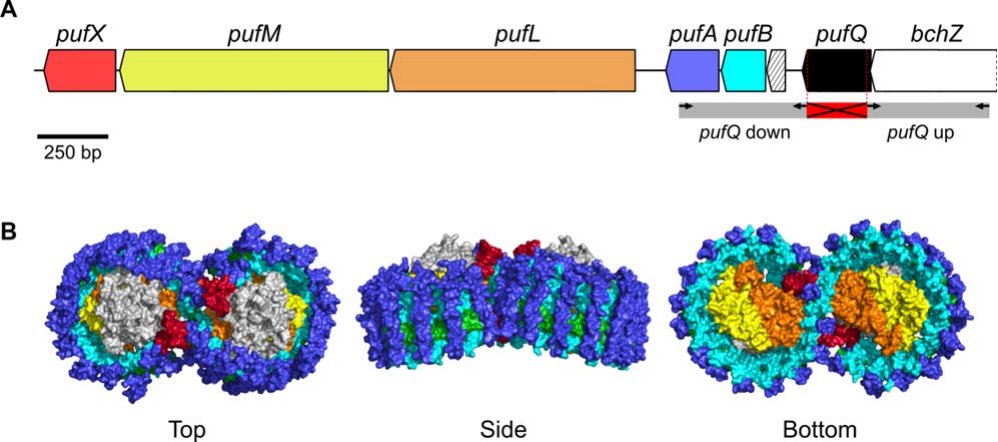
A. The *puf* operon of *Rba. sphaeroides*. The terminal 450 bp of the *bchZ* gene are shown (total length = 1476 bp). Regions cloned for *pufQ* knock out mutagenesis are indicated in grey; black arrows represent primers. The red, crossed‐out section indicates the region deleted in the Δ*pufQ* strain (nucleotides 10–227 were deleted leaving 1–9 and 228–234 in tact). The small hatched ORF represents *pufK*. B. The RC‐LH1‐PufX core complex of *Rba. sphaeroides* (Qian *et al*., 2013). LH1β and LH1α (light and dark blue, respectively) form a ring around a reaction center comprised RC‐L, RC‐M and RC‐H subunits (orange, yellow and grey, respectively). PufX (red) sits at the interface between the dimers. BChl molecules are shown in green. The colours of the polypeptide components correspond to the gene colour scheme in (A).

**Figure 2:**
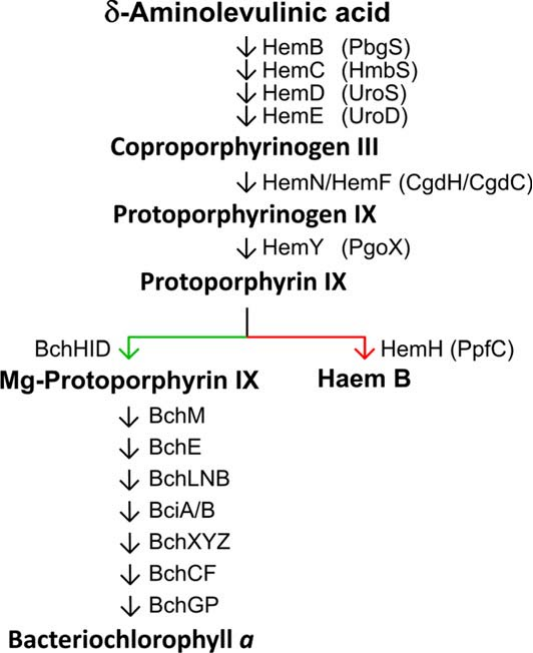
The Bacteriochlorophyll *a*/Haem B biosynthesis pathway of *Rhodobacter sphaeroides*. The Haem/BChl branchpoint is denoted by coloured arrows. Mg^2+^ insertion – green; Fe^2+^ insertion – red. Each arrow represents a modification to the previous molecule. Arrows are labelled with the relevant enzyme, and HemF and HemN are aerobic and anaerobic coproporphyrinogen oxidases, respectively. The protein abbreviations proposed recently by Dailey *et al*. (2017) are included in brackets.

The genes encoding the constituent proteins of the RC‐LH1‐PufX complex are situated within the photosynthesis gene cluster, which contains virtually all loci directly responsible for photosynthetic growth (Coomber and Hunter, 1989; Coomber *et al*., 1990; Naylor *et al*., 1999). The transition from non‐pigmented to fully pigmented cells is governed by oxygen and can take place in the dark (Cohen‐Bazire *et al*., 1957). The oxygen regulated *puf* operon consists of the *pufQ*, *B*, *A, L*, *M* and *X* genes encoding the LH1 α and β polypeptides, the RC L and M subunits and the PufX polypeptide respectively (Lee *et al*., 1989; Hunter *et al*., 1991; Fig. 1). At the other end of the photosynthesis gene cluster, the RC‐H subunit is encoded by *puhA* (Donohue *et al*., 1986; Williams *et al*., 1986 *;* Naylor *et al*., 1999). The assembly of RC‐LH1‐PufX and LH2 complexes depends on the availability of BChl, which shares a common biosynthetic pathway with haem up to protoporphyrin IX (Proto), at which point the pathway branches with the insertion of either Mg^2+^ or Fe^2+^ into the Proto macrocycle. Chelation of these metal ions, catalysed by magnesium chelatase (MgCH) and ferrochelatase (FeCH), respectively, commits tetrapyrrole biosynthesis to BChl and haem (Bollivar *et al*., 1994; Yaronskaya and Grimm, 2006). The haem/bacteriochlorophyll pathway is summarised in Fig. 2.

The metabolic versatility of *Rba. sphaeroides* requires a wide dynamic range for tetrapyrrole biosynthesis, in terms of the total flux that culminates in haem and BChl, as well as the balance between these pigments. Respiration requires haem for cytochromes whereas photosynthesis requires haems and, largely, bacteriochlorophylls. The FeCH/MgCH tetrapyrrole branchpoint must respond to oxygen levels and switch emphasis from haem to BChl, but the mechanism for allocation and regulation of flux down the haem/BChl branches has not been determined, despite the importance of *Rba. sphaeroides* as a model for tetrapyrrole biosynthesis for over seventy years (Van Niel, 1944; Lascelles, 1956; Cohen‐Bazire *et al*., 1957; Lascelles, 1959). We examined the *puf* operon, which contains two ORFs designated *pufQ* and *pufK* that do not encode structural components of the RC‐LH1‐PufX complex. It has been suggested that *pufK*, which contains a number of rare codons, has a role in ribosome stalling and serves to ‘gate’ entry of the ribosome into the *pufBA* gene pair (Gong and Kaplan, 1996). This, along with stem‐loop structures situated downstream of *pufBA*, serves to regulate the *pufBA* to *pufBALMX* transcript ratio allowing the LH1 components to be synthesised at higher levels than the less abundant RC components (Zhu *et al*., 1986; DeHoff *et al*., 1988). The *pufQ* gene sits at the start of the *puf* operon, sharing four bases with the terminus of *bchZ*, which encodes a subunit of the BChl biosynthesis enzyme chlorophyllide oxidoreductase (Nomata *et al*., 2006). Previous studies in both *Rba. sphaeroides* and the closely related bacterium *Rba. capsulatus* have demonstrated that the *pufQ* gene product is required for normal photosynthetic growth (Bauer and Marrs, 1988; Forrest *et al*., 1989; Hunter *et al*., 1991). Light‐harvesting complex levels were greatly reduced in mutants lacking *pufQ* and restoration of the *pufQ* gene in *trans* rescued the mutant phenotype. These observations led to speculation that PufQ has a regulatory role in BChl biosynthesis. Bauer and Marrs (1988) observed a similarity between the amino acid sequence of PufQ and RC transmembrane helices and proposed a pigment‐binding role for PufQ. Further studies suggested that the PufQ protein binds the BChl precursor chlorophyllide, that it may exert a stimulatory effect early in the BChl biosynthesis pathway, and that it may be directly involved with the assembly of LH1 and LH2 (Fidai *et al*., 1994, 1995; Gong *et al*., 1994). These studies draw conflicting conclusions, leaving an enigmatic role for the *pufQ* gene product, yet its presence in the photosynthesis gene cluster and its location at the start of the *puf* operon encoding essential light harvesting and RC complexes hint at an important role in photosystem biogenesis.

Here, we show that PufQ coordinates the BChl/haem biosynthetic pathways and the assembly of the RC‐LH1‐PufX complex. Interaction between PufQ and the haem biosynthesis enzyme FeCH is proposed to limit conversion of Proto to haem, making more Proto available to MgCH; thus, the haem/BChl branchpoint is directed toward BChl biosynthesis providing the cofactors that enter the assembly pathway for photosynthetic complexes.

## Results

### In‐frame deletion of *pufQ* results in cells with a low BChl phenotype that accumulates haem and coproporphyrin

**Figure 3:**
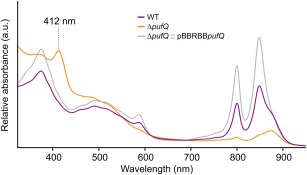
Absorbance spectra of WT, Δ*pufQ* and Δ*pufQ* :: pBBRBB*pufQ* cell‐free extracts. Spectra are normalised to the absorbance value at 680 nm.

**Figure 4:**
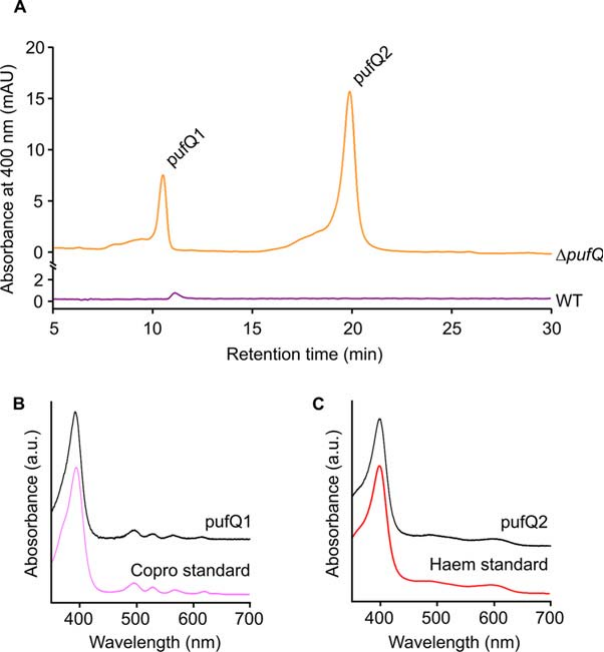
HPLC pigment analysis of the Δ*pufQ* mutant. Pigments were extracted in methanol and subjected to reverse phase HPLC analysis. A. HPLC chromatograms. B. Absorbance spectrum of the pufQ1 peak from (A) and a coproporphyrinogen III standard run under the same conditions (pink line). C. Absorbance spectrum of the pufQ2 peak from (A) and a haem B standard run under the same conditions (red line).

Markerless deletion of *pufQ* from the *pufQBALMX* operon of *Rba. sphaeroides* was achieved using the pK18mob*sacB* suicide vector system (Schäfer *et al*., 1994). Primers were designed in a way that left the upstream and overlapping *bchZ* gene intact, while ensuring that transcription through the new *pufBALMX* operon, driven by the oxygen‐regulated *puf* promoter (Hunter *et al*., 1991; Gregor and Klug, 1999), had not been disrupted. The deleted region is indicated in Fig. 1A. Δ*pufQ* mutant cells are orange in colour, as are many others harbouring mutations that abolish BChl biosynthesis. The *pufQ* gene was reintroduced to the Δ*pufQ* strain using the pBBRBB‐*Ppuf*_843–1200_ plasmid (Tikh *et al*., 2014). pBBRBB‐*pufQ* transconjugants displayed a WT‐like phenotype, with elevated levels of LH2 complexes (800 and 850 nm absorption), indicating that BChl biosynthesis has been restored and implying that the Δ*pufQ* phenotype does not arise from polar effects (Fig. 3; grey line). Absorption spectra of cell‐free extracts prepared from *ΔpufQ* mutant cells indicate low levels of the RC‐LH1‐PufX core complex (875 nm) and LH2 (850 nm) compared with WT (Fig. 3), demonstrating that the lowered BChl levels have affected assembly of the entire photosynthetic membrane. The Δ*pufQ* spectrum also has an absorption peak at 412 nm that is absent from the WT (Fig. 3). Reverse phase HPLC analysis of pigment extracts from the Δ*pufQ* mutant (Fig. 4) shows the accumulation of coproporphyrinogen III (Copro), a haem/BChl precursor and haem B, indicating that BChl biosynthesis is impaired before or at the magnesium chelation stage of BChl biosynthesis.

### Photosynthetic growth of *ΔpufQ* yields an intergenic suppressor mutant with an altered ferrochelatase

**Figure 5:**
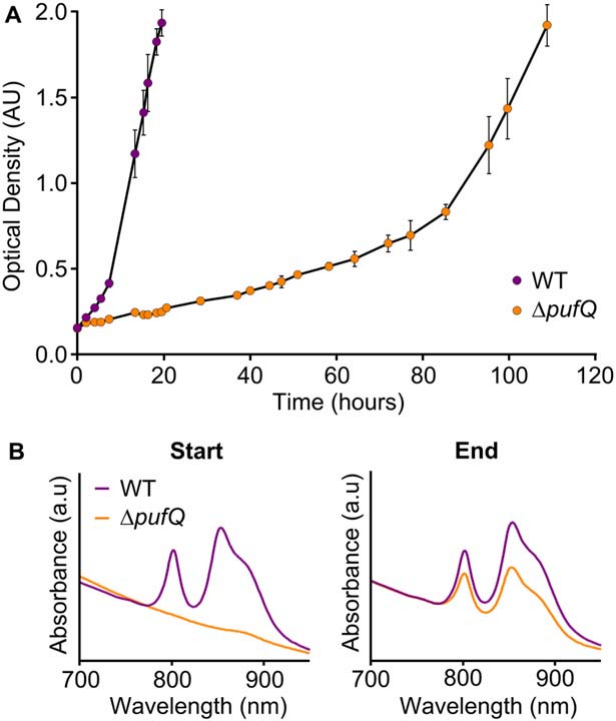
A. Photosynthetic growth curves of WT and Δ*pufQ* cells. Cells were grown in triplicate with moderate light (100 µE). Error bars represent the standard deviation of replicates. B. Absorbance spectra of WT and Δ*pufQ* cells at the starting (0 h) and finishing (WT: 19.5 h; Δ*pufQ*: 109 h) points of the growth curve experiment. Spectra represent one of three replicates; all replicates were checked and found to be similar. Spectra are normalised to 772 nm.

**Figure 6:**
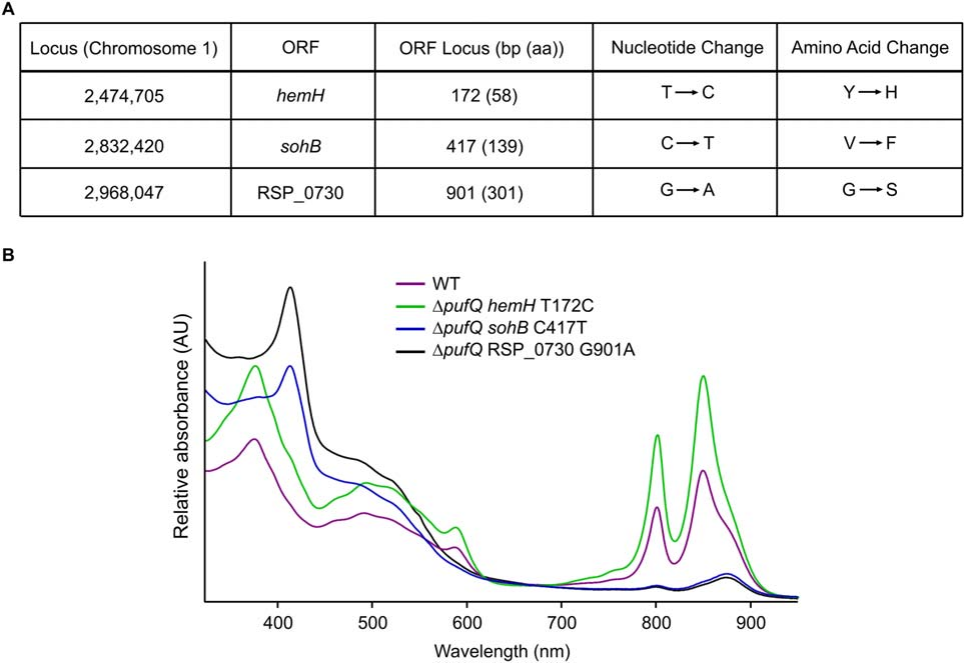
A. Summary of non‐silent point mutations within coding regions of the Δ*pufQ* PS_var_ mutant genome. ‘ORF locus’ represents the position within the gene (and protein) of the nucleotide (and amino acid residue) counted from the start codon of the coding strand. ORF – open reading frame; bp – base pair; aa – amino acid. B. Point mutations listed in (A) were individually introduced to the Δ*pufQ* background. Spectra of cell‐free extracts of each of these strains are shown. Spectra are normalised to 680 nm.

In order to investigate whether the small amounts of RC‐LH1‐PufX core complex present in the Δ*pufQ* mutant are enough to support photosynthetic growth under anaerobic conditions, photosynthetic growth curves were performed. Cells were cultured in small tubes filled, sealed and provided with constant illumination (50 µmol photons s^−1^ m^−2^). The results show that WT culture reaches an optical density of ∼ 2 within 20 h of inoculation (starting density = 0.15 AU) by which stage the Δ*pufQ* mutant had not yet doubled (OD_680_ = 0.25). However, after 110 h, the Δ*pufQ* strain had not only reached an OD of 2, but had also developed a WT‐like absorption profile implying that the cells had developed the ability to synthesise ‘normal’ levels of BChl and thus produce photosynthetic complexes (Fig. 5). These cells were streaked onto fresh agar plates and the resultant colonies retained a stable WT‐like phenotype under oxygen‐limited growth conditions in the dark, indicating the introduction of a suppressor mutation. Genomic DNA from this new photosynthetic variant of the Δ*pufQ* strain (Δ*pufQ* PS_var_) was isolated and sequenced. Analysis of the resulting data yielded three single‐base mutations situated in coding regions of the Δ*pufQ* PS_var_ genome that encoded amino acid changes, specifically within the *hemH* (ferrochelatase), *sohB* and RSP_0730 open reading frames; the mutations are summarised in Fig. 6A. In order to investigate which of these mutations rescued the mutant phenotype, the three different genomic point mutations were added individually to the Δ*pufQ* background using the pK18mob*sacB* suicide vector. The resultant strains were cultured and analysed by absorption spectroscopy (Fig. 6B). Introduction of the *sohB* or RSP_0730 point mutations appeared to have no impact on the phenotype of the cells whilst the *hemH* mutation yielded a phenotype similar to that of Δ*pufQ* PS_var_, demonstrating that the rescue observed in the original suppressor mutant was due to the point mutation within the ferrochelatase gene (Fig. 6B).

### Reduced activity of the mutant FeCH allows BChl biosynthesis in the photosynthetic Δ*pufQ* suppressor mutant

**Figure 7:**
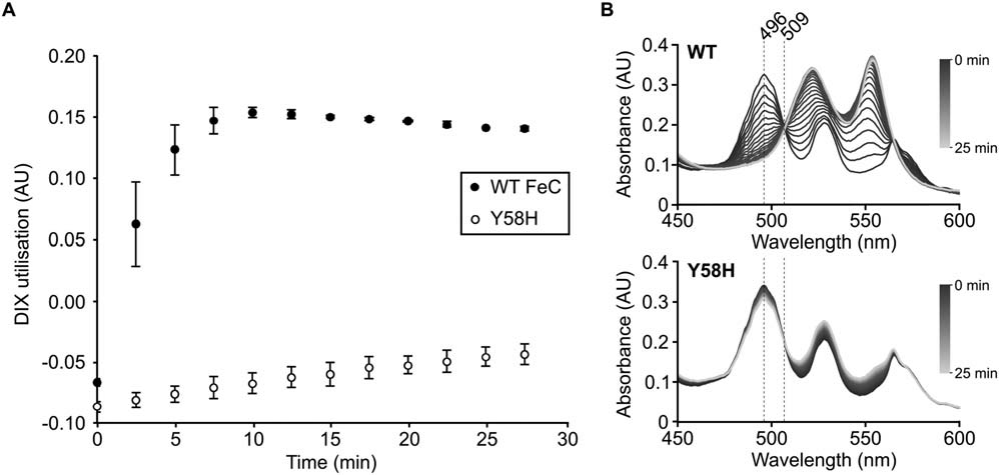
Characterisation of WT FeCH and Y58H mutant FeCH. Both enzymes were expressed heterologously in *E. coli* and purified by immobilised metal ion affinity chromatography. Spectrophotometric assays were performed in triplicate using 1 µM FeCH, 30 µM deuteroporphyrin IX and 100 µM CoCl_2_. A. Utilisation of DIX by FeCH. Values were calculated from the absorbance maxima at 496 nm using the isosbestic point at 509 nm as a reference. Error bars represent the standard deviation of the three replicates. B. Representative spectrophotometric datasets for WT and mutant (Y58H) FeCH assays, one replicate of three measured is shown. The wavelengths used to determine the values shown in (A) are highlighted with dashed lines.

The *hemH* gene from the Δ*pufQ* PS_var_ strain has a T to C point mutation at nucleotide 172, which changes a tyrosine to a histidine at residue 58 (Y58H) (Supporting Information Fig. S5, arrow). To determine the effects of this mutation on the activity of ferrochelatase, the cognate genes from both the WT and Δ*pufQ* PS_var_ strains were cloned and expressed heterologously in *Escherichia (E.) coli* (Supporting Information Fig. S1). Assays with the resultant purified recombinant protein showed that whilst the Δ*pufQ* PS_var_ version of the FeCH protein is active, the levels observed were substantially lower than for the WT protein (Fig. 7). We conclude that in the Δ*pufQ* PS_var_ strain, the reduced activity of the mutant FeCH serves to increase the availability of Proto to MgCH. This allows BChl biosynthesis to proceed at levels sufficient for normal photosystem assembly thereby rescuing the low BChl phenotype observed in the Δ*pufQ* mutant.

### FLAG immunoprecipitation of ferrochelatase yields an eluate containing PufQ; FLAG‐PufQ associates with ferrochelatase

**Figure 8:**
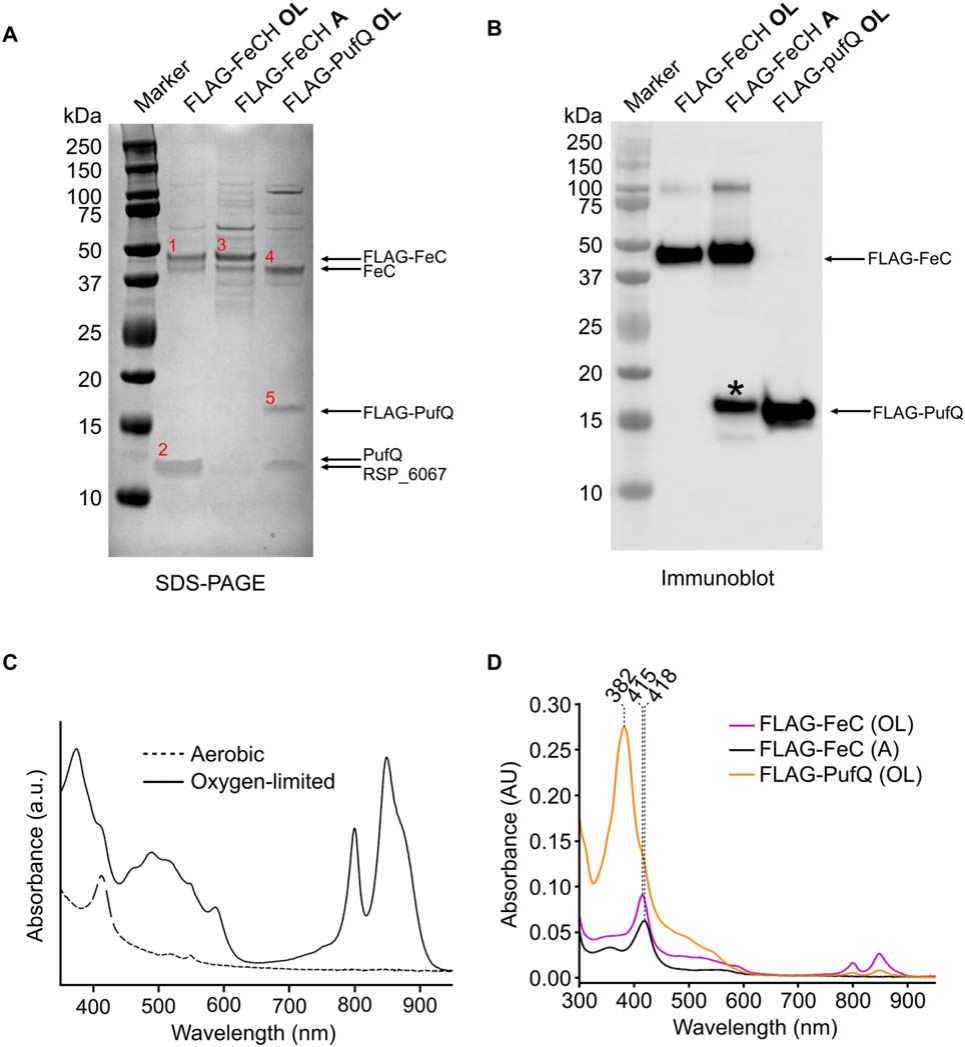
FLAG‐immunoprecipitation of FLAG‐tagged FeCH and PufQ. Lysates of both strains were applied to a column conjugated to immobilised anti‐FLAG antibodies. The eluates were analysed by SDS‐PAGE and either stained with Coomassie brilliant blue (A) or transferred to nitrocellulose membrane for immunoblotting then probing with an anti‐3xFLAG primary antibody (B). OL – oxygen limited; A – aerobic. The asterisk band represents the non‐specific cross reaction similar to that observed in Supporting Information Fig. S3. C. Absorbance spectra of the solubilised FLAG‐FeCH lysates prior to column application. D. Absorbance spectra of the FLAG‐immunoprecipitation eluates. Red numbers in (A) represent bands that were excised and analysed by nanoLC–MS/MS (Supporting Information Table S1).

To identify the interaction partners and near neighbours of PufQ, a strain of *Rba. sphaeroides* was created in which the genomic *pufQ* gene is modified with a sequence encoding an N‐terminal 3x FLAG‐tag (FLAG‐PufQ), enabling a series of FLAG‐immunoprecipitations using detergent‐solubilised cell lysates. Preliminary immunoprecipitation and mass spectrometry experiments with this strain indicated FeCH as a potential interaction partner for PufQ, so reciprocal immunoprecipitation experiments were performed with an N‐terminal 3x FLAG‐tag on FeCH (FLAG‐FeCH). Both FLAG‐tagged mutants retained a WT‐like phenotype indicating that the tagged proteins remained functional (Supporting Information Fig. S2). Given the strong influence of oxygen on transcription of the *puf* operon, the FLAG‐FeCH immunoprecipitation experiments were carried out on lysates prepared from cells grown under aerobic or oxygen‐limited conditions. The spectra of lysates derived from the aerobic and oxygen‐limited cultures are shown in Fig. 8C and demonstrate the suppression of photosynthetic apparatus under high oxygen conditions.

Analysis of the oxygen‐limited FLAG‐FeCH eluate by SDS‐PAGE and immunoblot (Fig. 8A and B) shows the presence of the FLAG‐FeCH bait protein, but few other potential interaction partners (Fig. 8A, lane 2). In order to further investigate the composition of the eluate, gel bands were excised and, following in‐gel tryptic digestion, were subjected to nanoLC‐MS/MS analysis. The results (Supporting Information Table S1A) show that PufQ is a prominent component of the eluate (Fig. 8A; Band 2), implying a PufQ‐FeCH interaction. In contrast, the eluate from aerobically grown FLAG‐FeCH cells does not appear to contain any PufQ and contains a wider range of interaction partners (Fig. 8A, lane 3), most of which are not present in either of the oxygen‐limited eluates. The FLAG‐PufQ eluate (Fig. 8A, lane 4) is similar in composition to that of the FLAG‐FeCH eluate (Fig. 8A, lane 2), containing both FeCH (Fig. 8A; band 4) and FLAG‐PufQ (Fig. 8A; band 5).

The FLAG‐FeCH and FLAG‐PufQ eluates both contain a protein of ∼ 13 kDa, manifested as a double band with PufQ in the FLAG‐FeCH OL elution (Fig. 8A, band 2), seen more obviously in the FLAG‐PufQ sample due to the size increase provided by the 3x FLAG‐tag. This band was identified by mass spectrometry as the protein product of the RSP_6067 gene (Supporting Information Table S1). In order to investigate this further a ΔRSP_6067 mutant was produced, which showed no discernible phenotype (data not shown). The reciprocal pulldowns in Fig. 6 show that FLAG‐PufQ immunoprecipitates with FeCH and FLAG‐FeCH immunoprecipitates with PufQ. These results indicate that PufQ interacts specifically with FeCH and that its binding influences the interaction profile of the FeCH protein.

The aerobic FLAG‐FeCH lane of the immunoblot contains a smaller (∼ 17 kDa) band that appears to cross react with the anti‐FLAG primary antibody (Fig. 8B; asterisk). As a check, immunoprecipitation experiments were carried out on solubilised lysates obtained from WT cells grown under aerobic and oxygen‐limited conditions; SDS‐PAGE showed that there were minimal contaminating bands demonstrating that the results observed with the FLAG‐tagged proteins are due to a specific interaction with the column. The 17 kDa band observed in Fig. 8B is also present in the aerobically grown WT eluate, indicating that this band arises as a result of a nonspecific cross‐reaction between either the primary or secondary antibody and a contaminating protein (Supporting Information Fig. S3).

**Figure 9:**
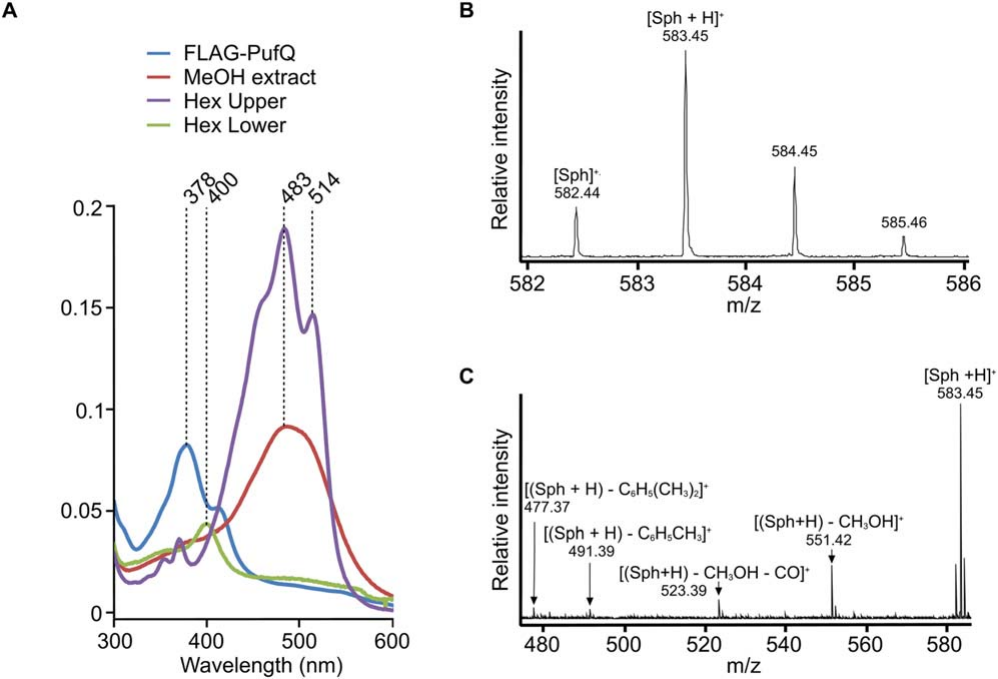
dentification of spheroidenone by absorbance spectroscopy and mass spectrometry. A. Absorbance spectra of pigment extracts from the FLAG‐PufQ eluate. The blue line represents the sample in buffer, the red line represents a methanol extract and the purple and green lines represent the upper and lower phases, respectively, after partitioning of the methanol extract in to hexane (1:1 ratio). B. The FLAG‐PufQ eluate was diluted fivefold with 50 mM ammonium acetate (AA) to reduce the β‐DDM concentration to 0.008%, subjected to five cycles of buffer exchange in AA and concentrated to a tenth of its original volume in a 10 kDa MWCO spin filter. The sample was analysed by electrospray mass spectrometry according to Canniffe *et al*. (2013). Mass spectrum showing the isotopic series for the spheroidenone radical cation ([Sph]^+^·) at *m*/*z* 582.44 and proton adduct ([Sph+H]^+^) at *m*/*z* 583.45. C. Product ion spectrum from the collision induced dissociation of ([Sph+H]^+^) showing neutral loss reactions diagnostic of the methoxy and carbonyl groups and methylated polyene chain of spheroidenone (Enzell *et al*., 1969).

Spectrophotometric analysis of the FLAG‐immunoprecipitation eluates (Fig. 8D) shows that the aerobic FLAG‐FeCH eluate contains a major peak at 418 nm, which is likely to be haem, the product of the FeCH catalysed reaction that has not been released from the enzyme active site. The oxygen‐limited FLAG‐FeCH spectrum contained a similar peak (415 nm), along with peaks at 800 nm and 850 nm characteristic of the BChls of the LH2 antenna complex and likely arising from contamination by some of this highly abundant antenna complex rather than specific binding to PufQ. LH2 absorption peaks were also observed in the spectrum of an eluate from a control FLAG‐immunoprecipitation using a lysate obtained from WT cells (Supporting Information Fig. S3C); here, there is no PufQ bait but a small amount of LH2, with its characteristic 800 and 850 nm absorption, binds non‐specifically to the FLAG column. The FLAG‐PufQ eluate spectrum contains the same B800 and B850 peaks, albeit at lower levels compared with the oxygen‐limited FLAG‐FeCH eluate, but also has a relatively large maximum at 382 nm. In order to evaluate this absorption feature further, pigments were extracted from the FLAG‐PufQ eluate and analysed by absorption spectroscopy and mass spectrometry. The results (Fig. 9) indicate that the peak arises from the carotenoid spheroidenone, which in the hexane‐solvated state has absorption maxima at 483 and 514 nm; the blue‐shifted 380 nm absorption could arise from a paired, parallel alignment of a pair of spheroidenone molecules (Fuciman *et al*., 2013).

In order to investigate the composition of the FLAG‐FeCH immunoprecipitation eluates further, proteins were digested in‐solution with trypsin and the resultant peptides analysed by mass spectrometry. The results show that FeCH interacts with both Copro oxidases (HemN, under oxygen‐limited conditions; HemF under aerobic conditions) and with protoporphyrinogen oxidase (HemY) under oxygen‐limited and aerobic conditions (Supporting Information Table S2).

### Ferrochelatase assays in the presence of PufQ

The results presented thus far indicate that the *pufQ* gene product interacts specifically with ferrochelatase. The *pufQ* gene sits at the start of the *puf* operon that encodes components of the RC‐LH1‐PufX core complex, the assembly of which has absolute requirement for BChl. It can therefore be hypothesised that in low oxygen conditions the *pufQ* gene product interacts with and downregulates FeCH in order to allow flux down the MgCH arm of the tetrapyrrole branchpoint, thus allowing BChl synthesis and subsequent RC‐LH1‐PufX assembly. This hypothesis is supported by the lowered activity of the recombinant Y58H variant of FeCH uncovered by the study of the Δ*pufQ* PS_var_ suppressor mutant in Figs 6 and 7, which allows some flux down the BChl biosynthesis pathway and thereby relieves the Δ*pufQ* mutant phenotype.

**Figure 10:**
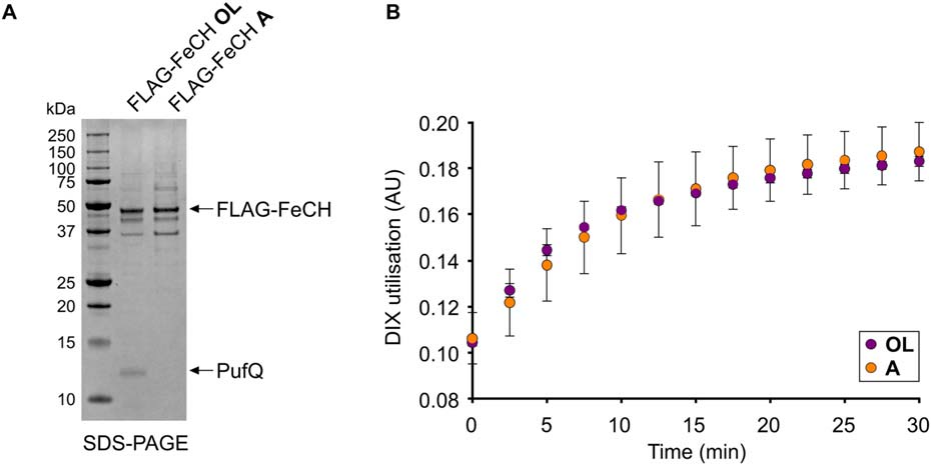
FeCH assays in the presence and absence of PufQ. A. FLAG‐immunoprecipitation of FeCH was carried out as in Fig. 7. The resultant eluates were analysed by SDS‐PAGE to confirm the presence or absence of PufQ. B. Assays were carried out as in Fig. 7, using 0.1 µM protein rather than 1 µM. The protein contents of the eluates, of which FeCH is the major component, were calculated using absorbance at 280 nm. The assays in B were performed with equal concentrations of FeCH.

In order to test this hypothesis, enzyme assays were carried out on FLAG‐FeCH immunoprecipitation eluates obtained from cells grown under high oxygen, which do not contain PufQ, and oxygen‐limited conditions, which do contain PufQ. SDS‐PAGE (Fig. 10A) confirms the respective absence and presence of PufQ in these eluates. The progress curves for both sets of assays demonstrate that the FLAG‐FeCH samples have similar activities (Fig. 9B), implying that the PufQ present in the oxygen‐limited sample has no effect on the activity of FeCH.

### Cellular levels of BChl and haem under aerobic and oxygen‐limited conditions demonstrate the need for nuanced regulation of the haem/BChl branchpoint

**Figure 11:**
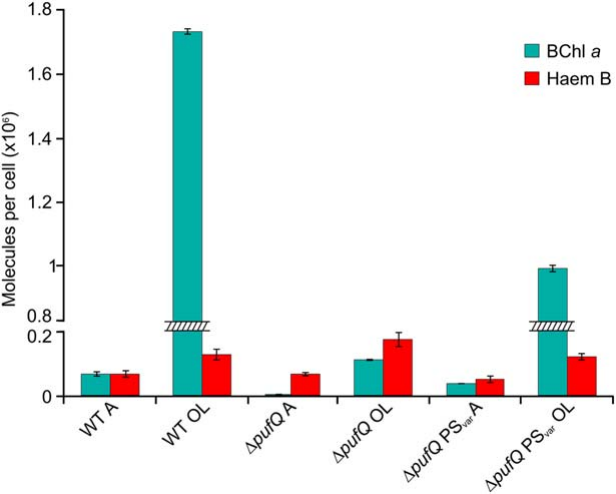
Quantification of cellular BChl and haem levels under aerobic and oxygen‐limited conditions. BChl was extracted from a known quantity of cells and analysed spectrophotometrically. Haem B was extracted from a known quantity of cells and analysed by reverse phase HPLC.

In order to further understand the cellular production of and demands for haem and BChl under different oxygen tensions, the levels of haem and BChl per cell were quantified from cultures grown under aerobic and oxygen‐limited conditions (Fig. 11). As would be expected the aerobic WT cells contain negligible levels of BChl when compared with the oxygen‐limited culture. This is also true of the Δ*pufQ* strain, although the oxygen‐limited culture contained only 6% of the WT levels of BChl per cell. This analysis shows that PufQ is essential for the production of normal levels of BChl; however, the large drop in BChl is not accompanied by a corresponding rise in the level of haem B, which in Δ*pufQ* OL increases only 36% in relation to the WT OL sample. As expected from Figs 5 and 6, Δ*pufQ* PS_var_ grown under oxygen‐limited conditions contains much more BChl per cell than the Δ*pufQ* mutant, although these levels are still 43% lower than in the WT. This demonstrates that while this strain is capable of photosynthetic growth, the lower activity of the Y58H variant of FeCH in this strain does not completely restore the BChl:haem balance to WT levels.

The haem B quantification results show an overall increase in haem levels per cell in oxygen‐limited cultures compared to aerobic cells, demonstrating that the shift from aerobic growth to oxygen‐limited cells that assemble photosynthetic complexes requires not only BChl production but also a net 95% increase in haem B levels. However, cellular haem production is not reflected in the levels of FeCH; Supporting Information Fig. S6 shows that aerobic (no BChl, no PufQ) cells contain approximately 10‐fold more FLAG‐FeCH protein than oxygen‐limited (pigmented, with PufQ) cells. Thus, cellular levels of FeCH, whether or not they are influenced by PufQ, do not correlate with haem production.

These results could explain the need for more nuanced regulation of the haem/BChl branchpoint; a simple switching of flux towards the BChl branch will not suffice because overall flux must increase towards protoporphyrin, and even though the majority must be routed down the BChl pathway, the FeCH branch must also produce more haem as well. Thus, lowering FeCH activity by binding PufQ would not work as a regulatory mechanism. Other ways of controlling the haem‐BChl branchpoint are discussed below.

## Discussion

The results in this study have demonstrated that PufQ controls the Fe^2+^/Mg^2+^ branchpoint in the tetrapyrrole pathway of *Rba. sphaeroides*, and in its presence, most of the flux is directed towards BChl, even though there is a near doubling of cellular haem levels as well. Thus, deletion of *pufQ* yields a mutant which synthesises only 6% of WT levels of BChl, resulting in cells with greatly impaired ability to assemble light‐harvesting and RC complexes. BChl biosynthesis is restored in the transconjugant strain Δ*pufQ* :: pBBRBB*pufQ*, and the presence of plasmid‐borne copies of *pufQ* appears to produce more LH2 complexes (800 and 850 nm absorption) than the WT, possibly because extra copies of PufQ divert even more flux down the BChl branch towards the photosystem assembly machinery. The discovery of an intergenic suppressor strain, followed by enzyme activity and immunoprecipitation experiments, identified the PufQ‐FeCH interaction as a means of controlling the allocation of tetrapyrroles to the haem and BChl pathways. However, a model for branchpoint regulation has to take into account the overall 14‐fold increase in cellular tetrapyrrole induced by oxygen‐limited growth, with preferential flow down the BChl branch, while also accounting for the increased production of cellular haem B.

### The accumulation of Copro in the *ΔpufQ* mutant suggests the presence of a switchable haem/BChl branchpoint enzyme supercomplex

The Δ*pufQ* strain has greatly lowered cellular BChl and accumulates the BChl/haem precursor Copro as well as haem B, implying that both the BChl and haem biosynthesis pathways are perturbed. The accumulated pigments give some insight into the role of PufQ. The pigment extraction utilised in Fig. 4 uses a solvent system designed for the extraction of haem/BChl precursors and it is not adequate for removal of haems from cytochromes. Hence, no haems are observed in the WT trace in Fig. 4A. The presence of haems in the Δ*pufQ* mutant extract (Fig. 3C) indicates an excess of ‘free’ haem within the cell which is not used for assembly of cytochromes. Accumulation of Copro in the Δ*pufQ* mutant (Fig. 4B) indicates a further destabilization of tetrapyrrole biosynthesis. Copro sits two steps before Proto in the haem/BChl biosynthesis pathway (summarised in Fig. 2), and the presence of this haem biosynthetic intermediate in the Δ*pufQ* mutant is consistent with the concept of a biosynthetic complex consisting of the aerobic Copro oxidase, Proto oxidase and FeCH. This is further supported by the fact that immunoprecipitation of FLAG‐FeCH retrieves an eluate containing both Copro oxidase and Proto oxidase (Supporting Information Table S2). In the thermophilic cyanobacterium *Thermosynechococcus elongatus* Proto oxidase and FeCH form a complex, although it does not include the aerobic Copro oxidase (Masoumi *et al*., 2008). A more extensive *Rhodobacter* complex that does include the aerobic Copro oxidase would convert Copro to haem, guided by a series of substrate channelling events, and accumulation of the haem product, as seen in the Δ*pufQ* mutant, could lead to accumulation of the initial substrate, Copro. The accumulation of this pigment in cells with perturbed tetrapyrrole biosynthesis was reported in early studies of haem and BChl biosynthesis (Lascelles, 1956), but it has never been explained. Proto, which under WT conditions is destined to become BChl, is apparently utilised by FeCH in the Δ*pufQ* mutant to make excess haem; this process is accompanied by an accumulation of Copro. The MgCH enzyme is still functional in this strain because some BChl is made, so the Δ*pufQ* mutant is apparently unable to deliver Proto to MgCH and thus regulate flux of the pathway along the BChl arm of the pathway. On this basis, PufQ could therefore be proposed as a positive effector of BChl biosynthesis with a similar role to the GUN4 protein found in chlorophyll *a* producing organisms (Larkin *et al*., 2003; Davison *et al*., 2005), perhaps ensuring delivery of Proto to MgCH. However, analysis of a suppressor mutant of the Δ*pufQ* strain (Δ*pufQ* PS_var_), generated by prolonged exposure to anoxygenic conditions, is consistent with a role for PufQ in controlling FeCH. The basis of the suppression was found to be a single base point mutation in *hemH*‐encoding FeCH. This altered FeCH has substantially lower activity compared to the WT enzyme and is sufficient to rescue the mutant phenotype and allow production of 57% the cellular levels of BChl compared with WT cells. This lower efficiency FeCH allows flux of Proto through the MgCH arm of the pathway in the absence of PufQ.

**Figure 12:**
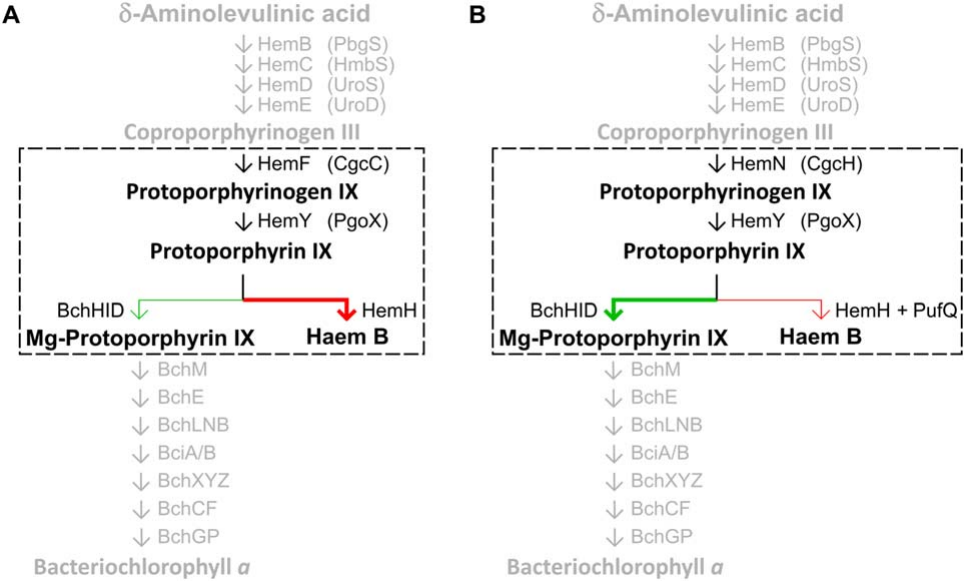
The proposed role of the PufQ protein in branchpoint regulation. A. Under high oxygen conditions, the *puf* operon is repressed and the haem arm of the pathway is favoured. B. Under oxygen‐limited conditions expression of the *puf* operon allows PufQ‐induced regulation of FeCH driving porphyrin flux towards bacteriochlorophyll. Perturbation of the enzymes within the dashed box, which could act in a concerted fashion, results in production of coproporphyrin.

These findings demonstrate that the PufQ protein has an essential role in regulation of the haem/BChl branchpoint. It appears that in the absence of PufQ, FeCH can utilise the available Proto pool to synthesise superfluous haem. However, if the FeCH is replaced with one that is less efficient, the balance is partially restored and unused Proto can proceed along the MgCH arm of the pathway. Figure 12 shows a schematic for the proposed involvement of PufQ in branchpoint regulation.

Although the immunoprecipitation experiments provide strong evidence for a specific interaction between PufQ and FeCH, a simple inhibition model for PufQ is not consistent with the FeCH activities observed in Fig. 9 and contradicts the increased levels of haem observed under oxygen‐limited conditions (Fig. 10). Although it initially seemed logical that PufQ inhibited FeCH, there are good reasons for this not to be the case. Chiefly, haem is found in multiple components of the photosynthetic electron transport chain. If PufQ were an efficient FeCH inhibitor, haem biosynthesis would be shut down in developing photosynthetic membranes preventing the formation of functional electron transport chain components. This conclusion is further reinforced by the haem B quantification data (Fig. 10), which shows that WT cells contain 95% more cellular haem B under conditions in which the photosynthetic apparatus develops than when it is repressed by O_2_. We speculate that PufQ could operate by limiting the access of FeCH to its Proto substrate, possibly restricting interactions between FeCH and neighbouring proteins that normally occur in the absence of PufQ. PufQ is a small (8.7 kDa) hydrophobic protein, containing a single transmembrane helix (Supporting Information Fig. S4A); we therefore suggest that the binding of PufQ to FeCH anchors it to a location within the developing membrane away from the available Proto pool, restricting the availability of Proto to FeCH, although further study would be required to confirm this.

The FLAG‐PufQ immunoprecipitation eluate contains an absorbance peak at 382 nm, which was not observed in either the WT control or either of the equivalent experiments using FLAG‐FeCH. It seemed likely that this would be a haem/BChl intermediate which bound to PufQ as part of its regulatory role. However, when pigments were examined by absorption spectroscopy and mass spectrometry it was found that this absorbance peak arises from the carotenoid spheroidenone. The absorbance maximum of the spheroidenone molecules in the FLAG‐PufQ eluate is blue‐shifted; this shift of carotenoid absorption to shorter wavelengths is a known phenomenon which occurs in aggregates known as H‐aggregates, in which the carotenoids align in a parallel orientation (Fuciman *et al*., 2013). The purpose of the carotenoids within the FLAG‐PufQ eluate is unclear although a role in photoprotection of the PufQ protein complex is one possible explanation, which should be investigated in further studies. There is a precedent for photoprotection by carotenoids in a biosynthetic complex; in the cyanobacterium *Synechocystis* the terminal enzyme in chlorophyll biosynthesis, chlorophyll synthase, forms a complex with the high light‐inducible protein HliD, the Ycf39 protein and the YidC/Alb3 insertase (Chidgey *et al*., 2014). It was subsequently shown that the purified Ycf39‐HliD subcomplex binds the carotenoid β‐carotene, which acts as a protective energy quencher of excited states in nearby chlorophyll‐*a* molecules (Staleva *et al*., 2015).

### Bioinformatic analysis of PufQ and FeCH

Secondary structure predictions of PufQ indicate that the protein contains a single hydrophobic transmembrane domain flanked by N‐ and C‐terminal soluble domains (Supporting Information Fig. S4A). Alignments of the PufQ protein sequences of five purple bacteria indicate that the transmembrane (TM) helix and C‐terminus are well conserved while the N‐terminus and the region between the TM helix and the C‐terminus are more divergent (Supporting Information Fig. S4B). The high level of identity observed at the C‐terminus, coupled with the fact that it is predicted to be situated on the cytoplasmic side of the membrane, indicates that this is likely to be the region that interacts with FeCH. The region between this well‐conserved domain and the TM helix is likely to have a more structural role given the levels of divergence between the different species. The high level of identity in the TM helix region could arise from some specific function such as pigment binding, as previously suggested (Bauer and Marrs, 1988). This idea is supported by the data shown in Fig. 8D, where the FLAG‐PufQ eluate is shown to contain a high level of spheroidenone. Although whether the PufQ protein binds these carotenoids directly, or indirectly via interactions with a carotenoid binding protein, remains to be seen. Finally, PufQ lacks the conserved residues indicative of a redox switch and so is unlikely to function in that manner.

Not all purple bacteria have a PufQ ortholog, which raises the question of how, if at all, they regulate their haem/BChl branchpoint. In order to evaluate any obvious differences between the FeCH proteins of PufQ containing organisms compared with non‐PufQ containing organisms, eight FeCH protein sequences were aligned. Five of these sequences were from organisms containing a *pufQ* gene (Q) and three were taken from organisms lacking a *pufQ* ortholog (nonQ) (Supporting Information Fig. S5). The results show that the FeCHs in PufQ containing bacteria tend to have longer N‐terminal domains before the area of high identity is reached (Supporting Information Fig. S5; residue 47). This region contains an area of homology found in the PufQ‐containing FeCHs but which is less well conserved among the non‐PufQ FeCHs (Supporting Information Fig. S5, boxed). It is possible that this region is the site of interaction with PufQ, although further work would be required to confirm this.

The residue at which the point mutation occurred in the Δ*pufQ* PS_var_ strain (Supporting Information Fig. S5, arrow) is conserved among the PufQ containing FeCHs but absent in all of the nonQ organisms. This is interesting given that this residue is important for the correct function of the enzyme in *Rba. sphaeroides*. It is possible that the nonQ organisms have lost this residue through evolution, effectively creating a less active FeCH and removing the need for branchpoint regulation. This could be investigated in future experiments in which the FeCHs of these organisms are produced heterologously, assayed and compared with those from the PufQ containing organisms. It is also possible that this residue is only important in the FeCHs of PufQ containing organisms and that the non‐pufQ FeCHs are as efficient. If this is the case, then a different mechanism for branchpoint regulation is likely to exist, which could also be a subject of future investigations.

## Concluding remarks

The results presented in this study demonstrate that the *pufQ* gene product is essential for haem/BChl branchpoint regulation in *Rba. sphaeroides* and that this regulation is achieved via interaction with FeCH.

Discovery of this regulatory role for the PufQ protein explains the position of *pufQ* at the start of the *puf* operon (Fig. 1). Under high oxygen conditions the *puf* promoter is repressed, preventing the expression of *pufQ*, as well as the genes encoding RC‐LH1‐PufX components. In oxygen‐limited conditions, the *puf* promoter drives expression of *pufQ* allowing BChl production and therefore assembly of photosynthetic complexes encoded by genes downstream of *pufQ*. This ensures that the production of light‐harvesting complex components is coordinated with the production of BChl.

PufQ‐FeCH complex formation is likely to be an important early step in photosynthetic membrane development. In *Rba. sphaeroides*, the FeCH enzyme is membrane associated (Dailey, 1982); it is likely that under high O_2_ conditions, FeCH is distributed throughout the cell providing haem for assembly of respiratory complexes. When O_2_ levels decrease to the threshold for *puf* operon expression, PufQ is produced and is inserted into the membrane. PufQ can then interact with nearby FeCH at the membrane surface, allowing the proto oxidase to deliver the proto substrate more directly to the Mg chelatase and thus creating an environment in which the photosynthetic apparatus can develop. If this is the case, PufQ could play a role in dictating the location of sites where membrane invagination is initiated. Supporting Information Figure S7 shows that PufQ is found in both precursor (UPB) and mature (ICM) membrane fractions, so PufQ might act as a useful marker for early developing membranes in future studies.

## Experimental procedures

### Standard buffers, reagents and media

All buffers and culture media were prepared as described in Sambrook *et al*. (1989), unless otherwise stated. All media and solutions were prepared using distilled water purified through a Milli‐Q system (Millipore, USA). Growth media and solutions used for DNA work were sterilised by autoclaving at 15 psi for a minimum of 20 min or by filtration through 0.2 μm filters. Heat‐labile solutions such as antibiotics and vitamins were only added to the culture medium once it had cooled to below 50°C.

### *E. coli* strains and plasmids

The *E. coli* strains used in this study were: JM109, S17‐1 (Simon *et al*., 1983) and Rosetta pLysS. JM109 cells were purchased from Sigma. S17‐1 cells were used for plasmid transfer into *Rba. sphaeroides* strains. Strains were grown in Luria–Bertani (LB) medium with antibiotics added when required. The following antibiotic concentrations (μg ml^−1^) were used: kanamycin 30; ampicillin 200; chloramphenicol 34.5. When grown in liquid, cells were agitated at 250 r.p.m.

### *Rba. sphaeroides* strains

*Rhodobacter sphaeroides* refers to wild‐type *Rba. sphaeroides* strain 2.4.1. Wild‐type and mutant strains were grown in M22+ medium (Hunter and Turner, 1988); 0.1% casamino acids were used to supplement liquid cultures. When required, cultures were supplemented with kanamycin to a final concentration of 30 µg ml^−1^. Stocks were stored in LB medium containing 50% glycerol (vol/vol) at −80°C.

### Oxygen‐limited growth of *Rba. sphaeroides*

Starter cultures consisting of 10 ml M22+ medium in 30 ml glass universals were incubated at 30°C with constant agitation at 150 r.p.m. These were used to inoculate larger cultures (80 ml in 125 ml flasks), and if larger volumes were required, the 80 ml cultures were subsequently used to inoculate 1.5 l of medium in 2 l flasks.

### Aerobic growth of *Rba. sphaeroides*

A total of 1 ml of a 10 ml oxygen‐limited starter culture was used to inoculate 50 ml of M22+ medium in a 250 ml baffled flask. Cells were grown at 30°C with constant agitation at 250 r.p.m. If larger cultures were required this culture was used to inoculate 500 ml of media in a 2 l baffled flask.

### Photosynthetic growth of *Rba. sphaeroides*

Anaerobic cultures of *Rba. sphaeroides* grown under photosynthetic conditions were exposed to 15 or 20 W MEGAMAN^®^ CFL bulbs to achieve the desired light intensity. Light intensity was measured in μmol photons s^−1^ m^2^ using a LI‐250A light meter equipped with a LI‐190 Quantum sensor (LI‐COR Biosciences, USA). One millilitre of oxygen‐limited culture was used to inoculate a full 30 ml universal of M22+ medium. A small magnetic stir bar was placed in the bottom of the bottle, and the culture was incubated at the desired light intensity, overnight with gentle agitation. This culture was used to inoculate either a 500 ml medical flat or a 1.2 l Roux culture bottle filled with M22+ medium and capped with a rubber bung. These cultures also contained a magnetic stir bar to provide gentle agitation.

### Manipulation of the *Rba. sphaeroides* genome

*Rhodobacter sphaeroides* gene deletion/modification was achieved using the pK18mob*sacB* suicide vector system as described in Mothersole *et al*. (2015). The primers used are listed in Supporting Information Table S3.

### Pigment extraction

A total of 1 ml of cell culture was pelleted by centrifugation, washed with 25 mM Tris pH 7.4 and resuspended in 1 ml extraction solvent. The cell‐solvent suspension was mixed thoroughly by vortexing and incubated on ice in the dark for 10 min. The mixture was clarified by centrifugation, yielding a supernatant containing extracted pigment. Extraction solvents were: 0.2% NH_3_ in methanol for BChl and BChl/haem precursors; acidified acetone (90:8:2, acetone:water:HCl) for haems.

### Reverse‐phase HPLC

Extracted pigments were loaded on to an Agilent‐1200 series HPLC system in conjunction with a Nova‐Pak C18 reverse phase column (4 μm particle size, 3.9 mm × 150 mm; Waters, USA).

### Separation of bacteriochlorophyll/haem precursors

Reverse‐phase HPLC was carried out using 350 mM ammonium acetate 30% methanol as solvent A and 100% methanol as solvent B. Pigments were eluted with a linear gradient of solvent B (65–75%, 35 min) followed by 100% solvent B at a flow rate of 1 ml min^−1^ at 40°C (Sobotka *et al*., 2011). Pigment content was monitored by absorbance (400, 433, 440, 632 and 663 nm) and fluorimetry (440 nm excitation and 670 nm emission). The coproporphyinogen III standard was extracted from the N1 mutant (Coomber *et al*., 1992); hemin was purchased from Sigma‐Aldrich (USA).

### Separation of haems

Reverse‐phase HPLC was carried out using 0.1% trifluroacetic acid in H_2_O as solvent A and 0.1% trifluroacetic acid in acetonitrile as solvent B. Pigments were eluted with a linear gradient of solvent B (25–100%, 30 min) at a flow rate of 1 ml min^−1^ at 40°C. Pigment content was monitored by absorbance at 400 nm (Sobotka *et al*., 2011).

### Genomic DNA sequencing and analysis

Genomic DNA isolation was performed using a MasterPure^®^ Complete DNA and RNA purification kit according to the manufacturer's instructions. The recovered genomic DNA was sequenced by BGI Tech (China). Contigs were assembled to the *Rba. sphaeroides* 2.4.1 genome using Geneious^®^ software. The same software was used to detect mismatches between the WT and mutant sequences.

### Overexpression and purification of His‐tagged ferrochelatase

Ferrochelatase genes were cloned into the multiple cloning site of pET14b using the *hemH* pET14b F and R primers listed in the primer table. The resultant plasmid was transformed into Rosetta pLysS BL21 *E. coli* cells. A total of 10 ml starter cultures were grown overnight (LB broth, 37°C, 250 r.p.m.) and used to inoculate 500 ml cultures (same conditions). Cultures were induced with isopropyl β‐d‐1‐thiogalactopyranoside once they had reached an OD_600_ of 0.6 and incubated overnight (20°C, 250 r.p.m.). Cells were harvested and lysed by sonication and the crude lysate clarified by centrifugation (60,000 *g*, 30 min). Proteins were separated by immobilised nickel affinity chromatography and His‐tagged ferrochelatase was eluted using buffer containing 400 mM imidazole.

### Spectrophotometric ferrochelatase assays

Assays were carried out in a Cary 60 UV–vis spectrophotometer (Agilent Technologies, USA) using the scanning kinetics programme. Ferrochelatase protein, deutroporphyrin IX and CoCl_2_ were added to assay buffer (25 mM Tris pH 7.4, 0.15% Tween 80) to the desired concentration in a final volume of 1 ml. CoCl_2_ was added immediately prior to initiation of the programme. Periodic absorbance scans between 450 and 600 nm were taken at 30 s intervals. The assay protocol was adapted from Cornah *et al*. (2002).

### FLAG immunoprecipitation

Cell cultures were harvested and resuspended in 1 mM Tris pH 7.4, 1 mM EDTA. Following incubation with lysozyme and deoxyribonuclease, cells were disrupted using a French pressure cell press at 18,000 psi. The lysate was clarified by centrifugation (60,000*g*, 30 min) and the pellet containing unbroken cells and debris was discarded. The supernatant, containing soluble proteins and membranes, was solubilised by the addition of *n*‐dodecyl β‐d‐maltoside (β‐DDM) to a final concentration of 1.5% with incubation at 4°C under gentle agitation for 60 min. The solubilised lysate was applied to a pre‐equilibrated 300 µl anti‐FLAG M2 affinity resin column. The column was washed with 30 column volumes of buffer containing 0.04% β‐DDM. Proteins were eluted by the addition of 75 µg FLAG‐peptide dissolved in wash buffer to the resin, which was removed from the column and transferred to a 1.5 ml sample tube. After incubation at 4°C for 60 min with gentle agitation FLAG‐tagged proteins were separated from the resin using a 0.22 µm Costar^®^ Spin‐X^®^ 0.22 µm filter.

### Immunoblot analysis of proteins

Proteins were separated by SDS‐PAGE and transferred to a nitrocellulose membrane. After blocking, membranes were incubated with rat anti‐FLAG primary antibody (Sigma) diluted to the appropriate concentration. After thorough washing, a horseradish peroxidise‐conjugated secondary antibody specific to rat IgG was added followed by further wash steps. Blots were imaged using WESTAR luminal solution (Cyanagen, Italy) in conjugation with an Amersham Imager 600RGB (GE Healthcare Life Sciences, USA).

### Quantification of cellular levels of BChl and haem B

Cultures to be analysed were harvested at exponential phase (OD_680_ = 1). Prior to pigment extraction the optical density of the cells to be pelleted was recorded. Cell number was calculated using the value of 1.1 × 10^9^ cells per optical density unit at 680 nm (Adams and Hunter, 2012). BChl *a* concentrations were calculated spectrophotometrically using an extinction coefficient of 60 mM^−1^ cm^−1^ (Cohen‐Bazire and Sistrom, 1966). Haem B levels were measured by integrating representative haem B absorbance peaks after reverse phase HPLC. Concentrations were calculated using a standard curve created by running a hemin standard using the same HPLC protocol in conjunction with an extinction coefficient of 170 mM^−1^ cm^−1^ (Collier *et al*., 1979).

### Identification of proteins in FLAG eluates

After elution from the FLAG resin, proteins were separated by SDS‐PAGE and stained with Coomassie Blue. Protein bands were excised from the gel and subjected to in‐gel digestion with trypsin according to Pandey *et al*. (2000). After extraction from the gel, tryptic peptides were analysed by nano‐flow liquid chromatography (Dionex Ultimate 3000 system, Thermo Scientific, USA) using a Targa 250 mm × 75 µm 5 µ C_18_ reverse‐phase column (Higgins Analytical, USA) and a linear gradient from 97% solvent A (0.1% formic acid, 3% acetonitrile) to 40% solvent B (0.1% formic acid, 97% acetonitrile) over 35 min at 300 nl min^−1^. On‐line mass spectrometry was performed using an Amazon ion trap instrument (Bruker Daltonics, Germany) programmed for automated dependent product ion scans with collision‐induced dissociation. Proteins were identified by database searching according to Mothersole *et al*. (2016).

## Author contributions

JWC and CNH designed experiments. JWC acquired and analysed all data with the exception of mass spectrometry analyses which were carried out by PJJ and MJD. JWC and CNH wrote the manuscript. All authors revised and approved the manuscript.

## Supporting information

Supporting Information
