# Supplementary material for: PufQ regulates porphyrin flux at the haem/bacteriochlorophyll branchpoint of tetrapyrrole biosynthesis via interactions with ferrochelatase
Source: Mol Microbiol. 2017 Nov 17;106(6):961–75. doi: 10.1111/mmi.13861 (PMC5725709; doi:10.1111/mmi.13861)
Supplement: Supplementary file 1 — Supporting Information [file MMI-106-961-s001.pdf]

Supplementary Information

PufQ regulates porphyrin flux at the haem/bacteriochlorophyll branchpoint of tetrapyrrole biosynthesis via interactions with ferrochelatase

Jack W. Chidgey[1], Philip J. Jackson[1,2], Mark J. Dickman[2] and C. Neil Hunter[1]*

[1]*Department of Molecular Biology and Biotechnology, University of Sheffield, Sheffield S10 2TN, United Kingdom*.

[2]*ChELSI Institute, Department of Chemical and Biological Engineering, University of Sheffield S1 3JD, United Kingdom*

*For Correspondence: Email c.n.hunter@sheffield.ac.uk. Tel.: +44 114 222 4191. Fax +44 114 222 2711

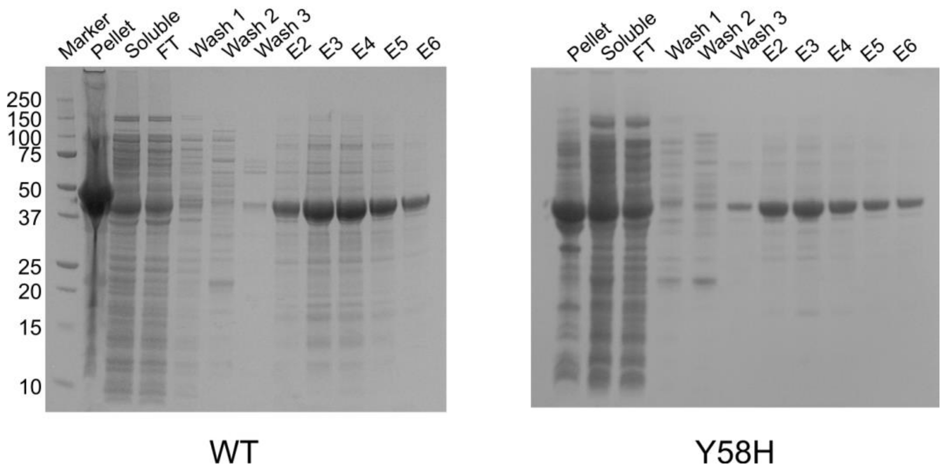

Figure S1. Purification of FeCH after heterologous expression in *E. coli.* Rosetta pLysS BL21 cells transformed with the appropriate plasmid were cultured and expression was induced by addition of Isopropyl β-D-1-thiogalactopyranoside. Harvested cells were lysed and the soluble fraction applied to a pre-equilibrated nickel column. After wash stages containing increasing imidazole proteins were eluted with buffer containing 0.4 mM imidazole. Pellet, insoluble fraction; Soluble, soluble fraction; FT, column flow through after application of soluble fraction; Wash 1, wash with binding buffer; Wash 2, wash with buffer containing 50 mM imidazole; Wash 3, wash with 100 mM imidazole; E2-6, elution fractions.

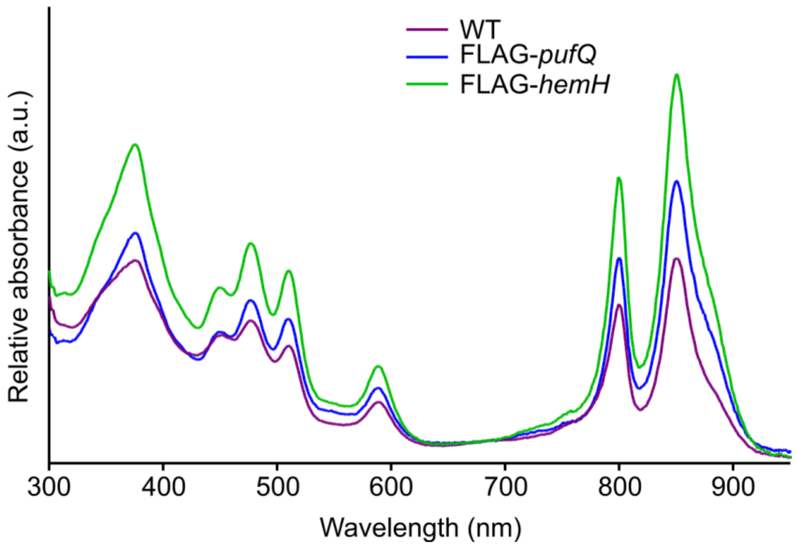

Figure S2. Absorption spectra of lysates obtained from FLAG-tagged strains grown under oxygen limited conditions. Spectra are normalised to the 680 nm.

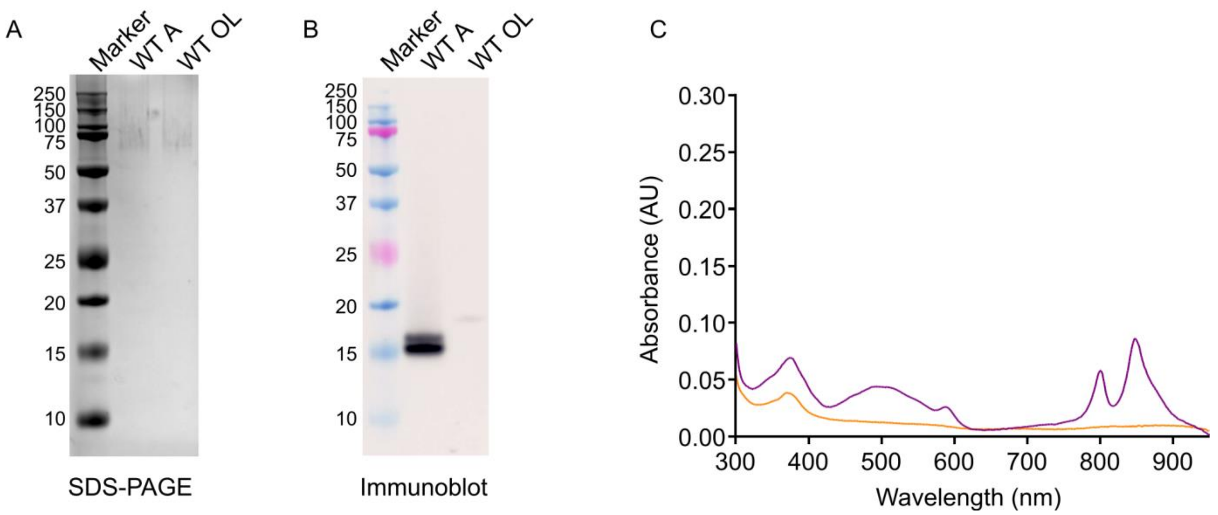

Figure S3. FLAG-immunoprecipitation WT control experiments. Lysates obtained from WT cells grown under aerobic and oxygen limited conditions were applied to a column containing immobilised antiFLAG antibodies exactly as described in Figure 8. Eluates were analysed by SDS-PAGE (**A**) and Immunoblot using 3xFLAG primary antibody (**B**). **C**. Absorption profile of the immunoprecipitation eluates shown in **A** and **B**. Orange line – aerobic, Purple line – oxygen limited.

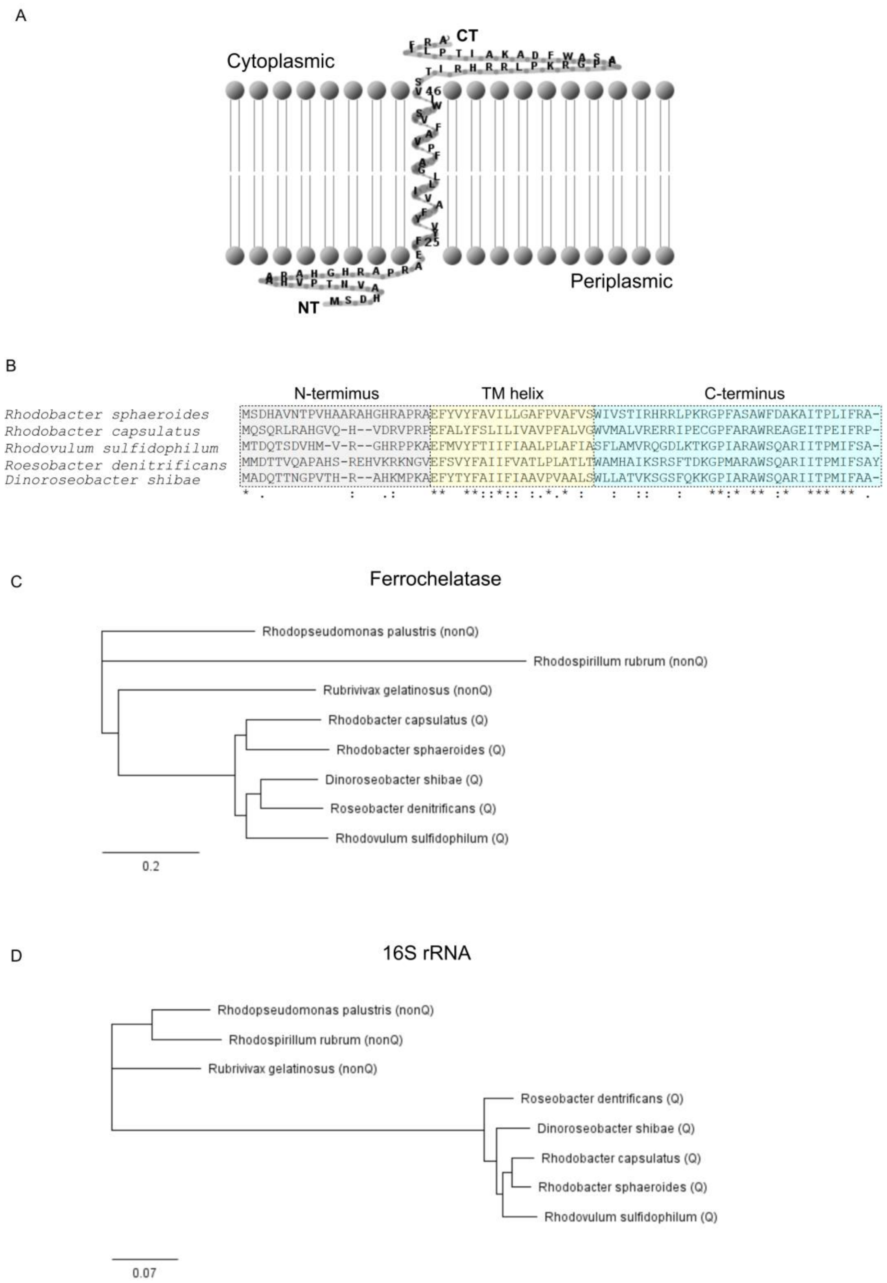

Figure S4. Bioinformatic analysis of PufQ and FeCH sequences. A. Predicted secondary structure of the PufQ protein of *Rba. sphaeroides*. Prediction generated using PRED-TMR (Pasquier et al., 1999), image generated using TMRPres2D software (Spyropoulos et al., 2004). B. Protein sequence alignments of five different PufQ proteins. Alignments were performed using T-coffee software (Di Tommaso et al., 2011), ***** - Fully conserved; **:** - Conservation of strongly similar amino acids; **.** – Conservation weakly similar amino acids. C. Phylogenetic tree of the protein sequences of ferrochelatases from eight purple bacteria, five of which contain pufQ homologues (Q) and three that lack a pufQ homologue (nonQ). Scale bar represents number of substitutions per base. D. Phylogenetic analysis of the 16S ribosomal RNA gene of the organisms in (C).

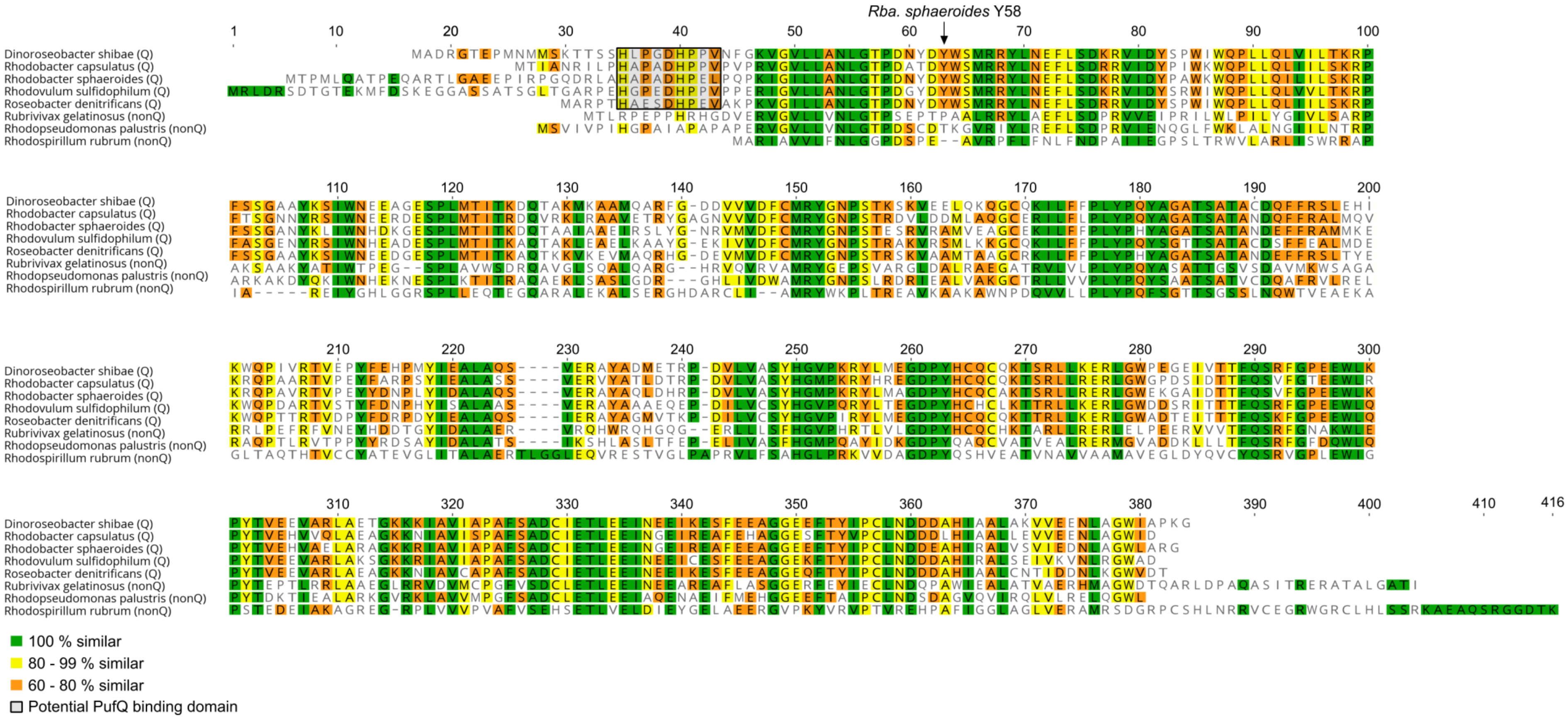

Figure S5. Alignment of the ferrochelatase protein sequences of five PufQ containing organisms (Q) and three that lack PufQ (nonQ). Residues are colour coded based on the similarity between the residues at the corresponding position in the other proteins (see key). The potential PufQ binding domain is boxed and highlighted in light grey. The residue which is mutated in the Δ*pufQ* PS_{var} mutant is indicated by an arrow.

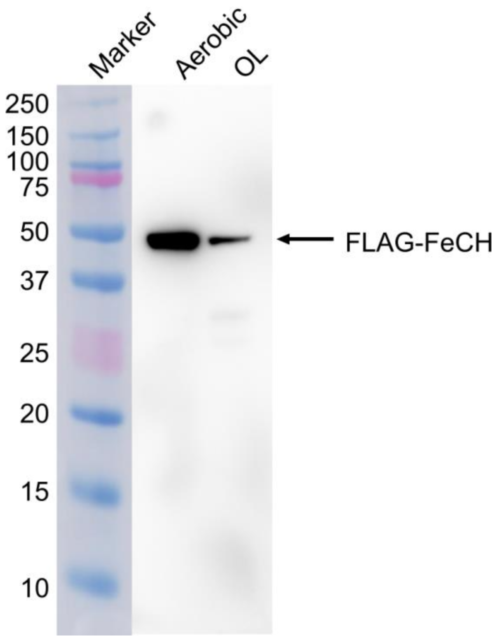

Figure S6. Analysis of the ferrochelatase content of cells grown under aerobic and oxygen limited conditions. FLAG-FeCH cell cultures were harvested at exponential phase. Cells were normalised to cell density before lysis. Equal amounts of lysate were separated by SDS-PAGE and probed by immunoblot using antiFLAG primary antibodies. Band intensity was analysed by ImageJ software, which demonstrated that the band in lane 2 (Aerobic) was roughly 10 fold more intense the band of similar size in lane 3 (OL).

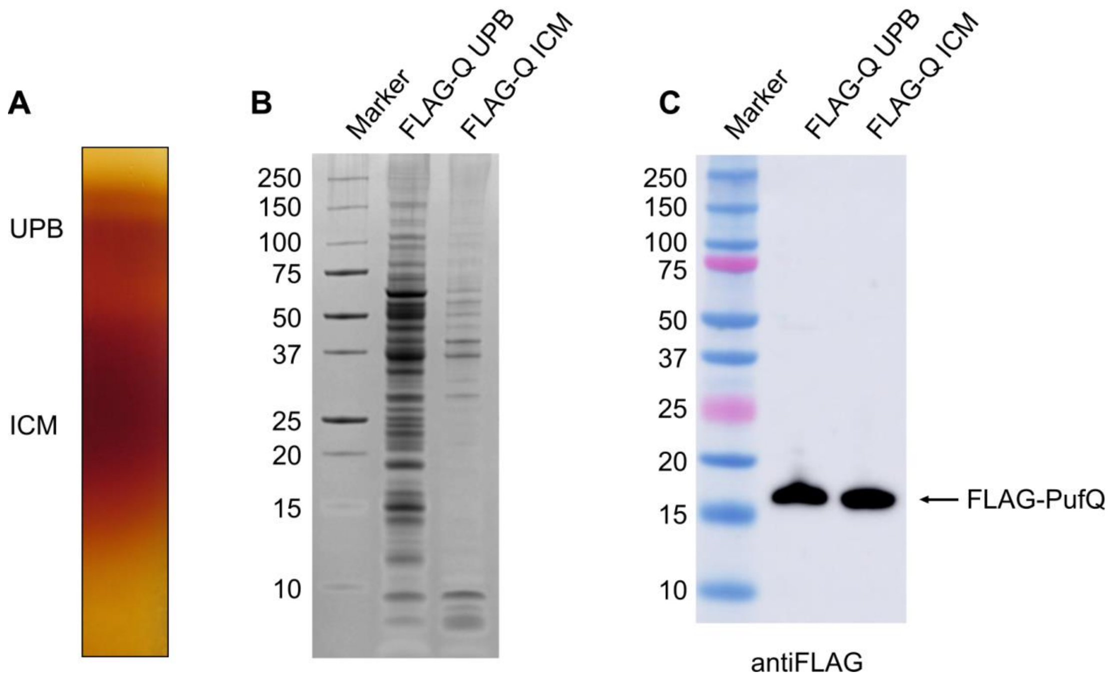

Figure S7. Separation of developing and mature photosynthetic membrane by sucrose density gradient centrifugation in the FLAG-PufQ (**A**) and FLAG-FeCH (**B**). Upper pigmented band (UPB) and Intracytoplasmic membrane (ICM) were analysed by SDS-PAGE and immunoblot.

Supplementary Table 1. Identification of proteins in eluates from FLAG immunoprecipitations

(A) SDS-gel lane: FLAG-FeCH **OL**

Band/Protein	Uniprot identifier	Score	Coverage (%)	Peptides detected
1/FeCH	Q3IYK5	849	53	R.TLGAEEPIRPGQDR.L
				R.LAHAPADHPELPQPK.I
				K.IGILLANLGTPDNYDYWSMR.R
				K.IGILLANLGTPDNYDYWSMR.R
				K.IGILLANLGTPDNYDYWSMRR.Y
				R.YLNEFLSDK.R
				R.YLNEFLSDKR.V
				R.VIDYPAWK.W
				K.RPFSSGANYK.L
				K.LIWNHDKGESPLMTITK.D
				K.LIWNHDKGESPLMTITK.D
				K.GESPLMTITK.D
				K.DQTAAIAAEIR.S
				R.VMVDFCMR.Y
				R.VMVDFCMR.Y
				R.TVPEYYDNPLYIDALAQSVER.A
				R.AYAQLDHRPDVLVASYHGMPK.R
				R.AYAQLDHRPDVLVASYHGMPK.R
				K.RYLMAGDPYHCQCAK.T
				R.YLMAGDPYHCQCAK.T
				R.YLMAGDPYHCQCAK.T
				R.LGWEK.G
				R.IAVIAPAFSADCIETLEEINGEIR.E
2/PufQ	Q3J1A1	263	44	M.SDHAVNTPVHAAR.A
				K.RGPFASAWFDAK.A
				R.GPFASAWFDAK.A
				K.AITPLIFR.A
				K.AITPLIFRA.-
2/RSP_6067	Q3J2T7	85	9	R.VALEDQAMR.R
				R.VALEDQAMR.R
				R.VALEDQAMRR.G

(B) SDS-gel lane: FLAG-FeCH **A**

Band/Protein	Uniprot identifier	Score	Coverage (%)	Peptides detected
3/FeCH	Q3IYK5	946	66	M.TPMLQATPEQAR.T
				R.TLGAEEPIRPGQDR.L
				R.LAHAPADHPELPQPK.I
				K.IGILLANLGTPDNYDYWSMR.R
				K.IGILLANLGTPDNYDYWSMR.R
				R.YLNEFLSDK.R
				R.YLNEFLSDKR.V
				R.VIDYPAWK.W
				K.RPFSSGANYK.L
				K.LIWNHDKGESPLMTITK.D
				K.GESPLMTITK.D
				K.DQTAAIAAEIR.S
				R.VMVDFCMR.Y
				R.TVPEYYDNPLYIDALAQSVER.A
				R.AYAQLDHRPDVLVASYHGMPK.R
				R.AYAQLDHRPDVLVASYHGMPK.R
				R.YLMAGDPYHCQCAK.T
				R.YLMAGDPYHCQCAK.T
				R.IAVIAPAFSADCIETLEEINGEIR.E
				R.EAFEEAGGEEFTYIPCLNDDEAHIR.A
				R.ALVSVIEDNLAGWLAR.G

(C) SDS-gel lane: FLAG-PufQ **OL**

Band/Protein	Uniprot identifier	Score	Coverage (%)	Peptides detected
5/PufQ	Q3J1A1	117	25	R.GPFASAWFDAK.A
				K.AITPLIFR.A
				K.AITPLIFRA.-
4/FeCH	Q3IYK5	552	54	R.TLGAEEPIRPGQDR.L
				R.LAHAPADHPELPQPK.I
				R.YLNEFLSDK.R
				R.YLNEFLSDKR.V
				R.VIDYPAWK.W
				K.RPFSSGANYK.L
				K.GESPLMTITK.D
				K.DQTAAIAAEIR.S
				R.VMVDFCMR.Y
				R.VMVDFCMR.Y
				R.VMVDFCMR.Y
				R.VRAMVEAGCEK.I
				R.TVPEYYDNPLYIDALAQSVER.A
				R.YLMAGDPYHCQCAK.T
				R.LGWEK.G
				R.IAVIAPAFSADCIETLEEINGEIR.E
				R.EAFEEAGGEEFTYIPCLNDDEAHIR.A
				R.ALVSVIEDNLAGWLAR.G

After SDS-PAGE analysis, protein bands were excised and subjected to in-gel trypsin digestion (Band number corresponds to the annotations in Figure 7). The extracted tryptic peptides were analysed by nanoLC-MS/MS and the resulting mass spectra used for proteomic database searching by Mascot (see Experimental Procedures). The scores are calculated from the inverse of the probability that an observed match is a random event with a significance threshold $p < 0.05$. The peptides detected are shown with their flanking residues separated by periods. Met residues that were detected as sulfoxides are underlined.

Supplementary Table 2. Identification of enzymes in the haem biosynthesis pathway in eluates from FLAG-FeCH immunoprecipitations.

(A) Oxygen-limited

Protein	Uniprot identifier	Score	Coverage (%)	Peptides detected
FeCH	Q3IYK5	17553	77	-.MTPMLQATPEQAR.T
				-.MTPMLQATPEQAR.T
				-.MTPMLQATPEQAR.T
				M.TPMLQATPEQAR.T
				R.TLGAEEPIRPGQDR.L
				R.LAHAPADHPELPQPK.I
				K.IGILLANLGTPDNYDYWSMR.R
				K.IGILLANLGTPDNYDYWSMR.R
				K.IGILLANLGTPDNYDYWSMRR.Y
				R.RYLNEFLSDK.R
				R.YLNEFLSDK.R
				R.YLNEFLSDKR.V
				R.RVIDYPAWK.W
				R.VIDYPAWK.W
				K.WQPLLQILILSK.R
				K.RPFSSGANYK.L
				K.LIWNHDK.G
				K.LIWNHDKGESPLMTITK.D
				K.LIWNHDKGESPLMTITK.D
				K.GESPLMTITK.D
				K.GESPLMTITK.D
				K.DQTAAIAAEIR.S
				R.VMVDFCMR.Y
				R.VMVDFCMR.Y
				R.VMVDFCMR.Y
				R.VMVDFCMR.Y
				K.ILFFPLYPHYAGATSATANDEFFR.A
				R.TVPEYYDNPLYIDALAQSVER.A
				R.AYAQLDHRPDVLVASYHGMPK.R
				R.YLMAGDPYHCQCAK.T
				K.GAIDTTFQSVFGPEEWLKPYTVEHVAELAR.A
				R.IAVIAPAFSADCIETLEEINGEIR.E
				R.ALVSVIEDNLAGWLAR.G
				R.ALVSVIEDNLAGWLARG.-
HemN	P33770	71	12	M.TNIALLQSLGLFDAR.V
				R.VAIFGYAHVPWMAK.R
				R.HELANLAAR.L
				R.AIEMIMCDFFLDLPALR.A
HemY	Q3J6D5	43	9	R.ALSEGMMALAAGEPR.T
				K.AFEINPK.H
				K.QSPDLIPAAAMAAR.S
				R.ALGDIVAR.H

(B) Aerobic

Protein	Uniprot identifier	Score	Coverage (%)	Peptides detected
FeCH	Q3IYK5	36403	84	-.MTPMLQATPEQAR.T
				-.MTPMLQATPEQAR.T
				-.MTPMLQATPEQAR.T
				M.TPMLQATPEQAR.T
				M.TPMLQATPEQAR.T
				R.TLGAEEPIRPGQDR.L
				R.LAHAPADHPELPQPK.I
				K.IGILLANLGTPDNYDYWSMR.R
				K.IGILLANLGTPDNYDYWSMR.R
				K.IGILLANLGTPDNYDYWSMRR.Y
				K.IGILLANLGTPDNYDYWSMRR.Y
				R.RYLNEFLSDK.R
				R.YLNEFLSDK.R
				R.YLNEFLSDKR.V
				R.RVIDYPAWK.W
				R.VIDYPAWK.W
				R.VIDYPAWKWQPLLQILILSK.R
				K.WQPLLQILILSK.R
				K.WQPLLQILILSKRPFSSGANYK.L
				K.RPFSSGANYK.L
				K.LIWNHDK.G
				K.LIWNHDKGESPLMTITK.D
				K.LIWNHDKGESPLMTITK.D
				K.GESPLMTITK.D
				K.GESPLMTITK.D
				K.DQTAAIAAEIR.S
				K.DQTAAIAAEIRSLYGNR.V
				R.VMVDFCMR.Y
				R.VMVDFCMR.Y
				R.VMVDFCMR.Y
				R.VMVDFCMR.Y
				R.AMVEAGCEK.I
				K.ILFFPLYPHYAGATSATANDEFFR.A
				R.TVPEYYDNPLYIDALAQSVER.A
				R.AYAQLDHRPDVLVASYHGMPK.R
				R.AYAQLDHRPDVLVASYHGMPK.R
				R.AYAQLDHRPDVLVASYHGMPKR.Y
				K.RYLMAGDPYHCQCAK.T
				K.RYLMAGDPYHCQCAK.T
				R.YLMAGDPYHCQCAK.T
				R.YLMAGDPYHCQCAK.T
				R.ERLGWEK.G
				K.GAIDTTFQSVFGPEEWLKPYTVEHVAELAR.A
				K.RIAVIAPAFSADCIETLEEINGEIR.E
				R.IAVIAPAFSADCIETLEEINGEIR.E
				R.ALVSVIEDNLAGWLAR.G

				R.ALVSVIEDNLAGWLARG.-
HemF	Q3J026	92	13	R.ILAAFEGLEDAATGGSPADAAPGR.F
				R.AVGEAFLPAFVPLVER.R
HemY	Q3J6D5	193	29	M.LWSLIK.I
				R.ALSEGMMALAAGEPR.T
				K.YLGQNAMTTLLNAQAAQQAGDSRR.A
				K.LDEGDTDTALALAQK.A
				K.AFEINPK.H
				K.HSETQDILLK.L
				R.DAVLALQTAK.D
				R.EAAILANK.Q
				K.QSPDLIPAAAMAAR.S
				R.TLTAIHPDHDETR.M
				R.ALGDIVAR.H
				R.ALTIMAAVER.G

Proteins in the FLAG eluates were precipitated, redissolved in 8M urea and subjected to in-solution digestion with a combination of endoproteinase Lys-C and trypsin. The peptides were analysed by nanoLC-MS/MS and the resulting mass spectra used for proteomic database searching by Mascot (Hollingshead *et al*., 2016). In addition to FeCH and other co-isolated proteins, coproporphyrinogen-III oxidase (HemN and HemF) and protoporphynongen III (HemY) were identified. The scores are calculated from the inverse of the probability that an observed match is a random event with a significance threshold $p < 0.05$. The peptides detected are shown with their flanking residues separated by periods. Met residues that were detected as sulfoxides are underlined.

Name	Sequence	Restriction Site
Δ*pufQ* UF	CCGGAATTCTTGTACTTGGCGGCAGAGATTTCCAGC	EcoRI
Δ*pufQ* UR	GCGCTCTAGAGCCTGACCGCAGGTCAGGTTG	XbaI
Δ*pufQ* DF	GCGCTCTAGAATCGCTCATTGGTTCTCTCCCTTCCTC	XbaI
Δ*pufQ* DR	CCCCAAGCTTCGATCAACGAGAAGATTTACCTTGCGGAA	HindIII
pufQ BBRBB F	GCCGAGATCTATGAGCGATCATGCCGTCAACACGC	BglII
pufQ BBRBB R	CCGCCTCGAGTCAGGCGCGGAAAATCAGCGGCGT	XhoI
sohB C417T F	GCGCTCTAGAATCTTGACGAGCATCAGAGACC	XbaI
sohB C417T R	GCGCAAGCTTATCCGGCTTCAGGGTGTGATC	HindIII
RSP_0730 G901A F	GCGCTCTAGAGCTGATGTTCGGCTCGATCTG	XbaI
RSP_0730 G901A R	GCGCAAGCTTCGCATCTCGATCAGGGCCG	HindIII
hemH T172C F	CGCGGAATTCAGTTTCGAGATCATCTGCCTCAC	EcoRI
hemH T172C R	CCCCAAGCTTGCATAATGAGGATAGAGCGGGAAGAAG	HindIII
hemH pET14b F	CCCCATATGACGCCGATGCTGCAAGCG	NdeI
hemH pET14b R	GCGCCTCGAGGCCGCGCGCGAGCCAG	XhoI
FLAG-*hemH* UF	CGCGTCTAGACGCTTGTCCGGGAGGTCCG	XbaI
FLAG-*hemH* UR	CGCGATTAATTCCGCTTCCAGACATTCAATCCGTC	VspI
FLAG-*hemH* DF	CGCGGCGGCCGCAATGACGCCGATGCTGCAAGCG	NotI
FLAG-*hemH* DR	CGCGGTCGACCTTGGTGATCGTCATGAGCGGG	SalI
FLAG-*pufQ* UF	CGCTCTAGAACGGCCCGCAACCCTCTTTCATCG	XbaI
FLAG-*pufQ* UR	GCGCCATATGTGGTTCTCTCCCTTCCTCTCGCC	NdeI
FLAG-*pufQ*DF	TCTAGAGCGGCCGCATGAGCGATCATGCCGTCAACACGCC	NotI
FLAG-*pufQ* DR	GGCCGTCGACGTCCGTAAGACCTGTGTAGCCCAGG	SalI

Supplementary Table 3. Primers used in this study. The indicated restriction site is underlined.

Supplementary references:

Di Tommaso, P., Moretti, S., Xenarios, I., Orobitg, M., Montanyola, A., Chang, J.M., *et al.* (2011) T-Coffee: a web server for the multiple sequence alignment of protein and RNA sequences using structural information and homology extension. *Nucleic Acids Res* **39**: W13-17.

Hollingshead S, Kopecna J, Armstrong DR, Bucinska L, Jackson PJ, Chen GE, Dickman MJ, Williamson MP, Sobotka R & Hunter CN (2016). Synthesis of chlorophyll-binding proteins in a fully segregated *Δycf54* strain of the cyanobacterium *Synechocystis* PCC 6803. *Front Plant Sci* 7: Article 292.

Pasquier, C., Promponas, V.J., Palaios, G.A., Hamodrakas, J.S. and Hamodrakas, S.J. (1999) A novel method for predicting transmembrane segments in proteins based on a statistical analysis of the SwissProt database: the PRED-TMR algorithm. *Protein Eng* **12**: 381–385.
